# Supplementary material for: Complexome profiling of the Chlamydomonas psb28 mutant reveals TEF5 as an early PSII assembly factor
Source: Plant Cell. 2025 Mar 17;37(6):koaf055. doi: 10.1093/plcell/koaf055 (PMC12164590; doi:10.1093/plcell/koaf055)
Supplement: koaf055_Supplementary_Data [file koaf055_supplementary_data.zip › psb28_interactive.zip!/plots/intensity/CYN38_Cre03.g189800.t1.2.html]

|  |  |
| --- | --- |
| **Identifier** | Cre03.g189800 |
| **Trivial name** | CYN38 |
| **Euclidean distance** | 321740.22 |
| **Average intensity sum (WT)** | 7324.9 || **Average intensity sum (Mut)** | 26041.7 |
| **Adjusted euclidean distance** | 12.35 |
| **MapMan annotation** | protein.folding.immunophilins (IMM).cyclophilins protein.folding |
| **p-value** (Welch test) of intensity sums | 0.05653 |
